# Socio‐economic factors do also matter: comments on the article “Can climatic factors explain the differences in COVID-19 incidence and severity across the spanish regions?: an ecological study”

**DOI:** 10.1186/s12940-021-00701-6

**Published:** 2021-02-18

**Authors:** Arthit Phosri, Yang Cao, Mariko Harada Sassa, Kouji H. Harada

**Affiliations:** 1 Department of Environmental Health Sciences, Faculty of Public Health, Mahidol University, 10400 Bangkok, Thailand; 2 Department of Preventive Medicine, St. Marianna University School of Medicine, 2168511 Kawasaki, Japan; 3 Department of Health and Environmental Sciences, Kyoto University Graduate School of Medicine, Yoshida Konoe, Sakyo 6068501 Kyoto, Japan

**Keywords:** Socio‐economic factors, Climatic factors, COVID-19, Ultraviolet radiation, Statistical analyses

## Abstract

A report published in this journal showed an inversely significant association between ultraviolet radiation (UVR) before the pandemic and cumulative COVID-19 cases in Spain. The analyses employed several meteorological factors, but socio-economic factors were not included. We examined the associations of COVID-19 cases with selected factors and found a significance on gross domestic product per capita (p = 0.037 by Spearman’s correlation). Hence, simple regression analyses of UVR would be confounded with regional difference in economic activities. In addition, we raised several questions for limitations due to the study design and analyses.

**Dear Editor**

Recent research article published in this journal demonstrated that the number of COVID-19 cases associated with ultraviolet radiation (UVR) in Spain [1]. It is possible that UVR can determine Vitamin D status and may modify the susceptibility to SARS-CoV-2. UVR also disinfects SARS-CoV-2 as shown by several experimental studies [2, 3]. However, it was an ecological study with limited evidence, and as authors stated as a limitation, there may be potential biases and the findings must be confirmed with individual data analyses. We would also comment further points that there is a large uncertainty regarding to the results.

Firstly, even an ecological study reporting correlation between specific weather variables and cumulative incidence of COVID-19 should consider possible confounders. Since the variation of the cumulative incidence of COVID-19 could be explained by other variables as well, such as socioeconomic, demographic factors, and healthcare policy. Such variables were not taken into account by authors. We know that there is a difference in population densities and economic disparities among the regions of Spain included in the study [4]. Economic activity is apparently related to human activity and the probability of human contact to infected individuals is a major factor in the transmission of infectious diseases. Indeed, previous world-wide comparison showed correlations between COVID-19 cases and gross domestic product (GDP), and GDP per capita [5]. Then, we examined the association between number of COVID-19 cases in Spain given in the article with GDP per capita in the regions, and a significant correlation was obtained similarly to UVR (*p* = 0.037 by Spearman’s correlation; Fig. 1). Therefore, authors could not conclude that a weather variable, UVR predominantly influences COVID-19 incidence and severity unless other significant variables are included.

Secondly, Fig. 2 of the original article [1] showed that the community with higher temperature was also correlated with lower cumulative incidence of COVID-19. However, it could not imply that people residing in community with high temperature will have lower risk of COVID-19 infection since the community-level data were used in the study instead of individual data. At least, they need to investigate the relationship between specific weather factors and daily or weekly COVID-19 incidences separately in each community.

Thirdly, the authors also mentioned that pattern of the association between humidity and COVID-19 incidence in the study was inconsistent with a previous preprint by Wang et al., 2020 [6]. The difference could be due in part to the statistical method of adjustment of covariates.

We believe that environmental factors determine the human susceptibility to diseases and behaviors especially to human contacts. Nonetheless, the methodology used in this study was insufficient to conclude that specific weather variables influence the incidence of COVID-19 in Spain, at least as hypotheses. Thus, the appropriate statistical method should be further applied to obtain the possible associations.

**Fig. 1:**
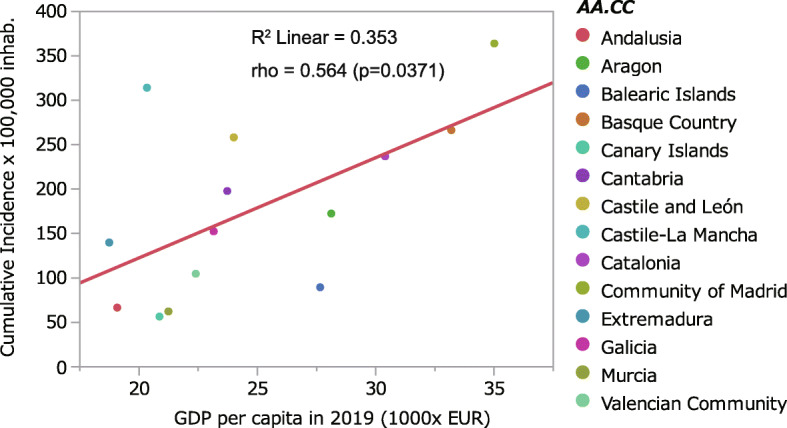
Relationship between gross domestic product per capita in 2019 and cumulative incidence of SARS-CoV-2 infection across the Spanish AA.CC

## Data Availability

Data and material sharing are not applicable to this letter.
